# Translational Preclinical Research may Lead to Improved Medical Management of Non-Arteritic Anterior Ischemic Optic Neuropathy

**DOI:** 10.3389/fneur.2014.00122

**Published:** 2014-07-11

**Authors:** James D. Nicholson, Hana Leiba, Nitza Goldenberg-Cohen

**Affiliations:** ^1^The Krieger Eye Research Laboratory, Felsenstein Medical Research Center, Petach Tikva, Israel; ^2^Sackler Faculty of Medicine, Tel Aviv University, Tel Aviv, Israel; ^3^Department of Ophthalmology, Kaplan Medical Center, Rehovot, Israel; ^4^Hebrew University Hadassah Medical Center, Jerusalem, Israel; ^5^Pediatric Ophthalmology Unit, Schneider Children’s Medical Center of Israel, Petach Tikva, Israel

**Keywords:** ischemic optic neuropathy, animal models, treatment, AION, non arteritic

Ischemic optic neuropathy is a major cause of significantly reduced vision (1–3). It may be arteritic (AAION) or non-arteritic (NA-AION), anterior or posterior. NA-AION, the most common form (1–4), is characterized clinically by acute, monocular, painless visual loss with optic disk swelling (5). It is attributed to an ischemic insult to the optic nerve followed by an inflammatory reaction (6, 7). The optic nerve head (ONH), is sensitive to minute changes in blood flow and therefore very susceptible to vascular insufficiencies due to altered autoregulation, vasospasm, and systemic vascular disease. However, the process leading to non-arteritic anterior ischemic optic neuropathy (NA-AION) appears to be complex and multi-factorial (8), and the exact cause is still unknown.

## Risk Factors Associated with NA-AION

### Systemic conditions

Non-arteritic anterior ischemic optic neuropathy usually occurs in the presence of an underlying vascular disease such as hypertension, diabetes, atherosclerosis, hypercholesterolemia, and regional vascular endothelial disorders, all of which predispose patients to ischemic stroke. In some cases, NA-AION is the first sign of these underlying conditions.

The role of a thrombotic tendency in NA-AION is controversial. Several studies associated elevated levels of plasma homocysteine and lipoprotein (a) and decreased levels of vitamin B6 with NA-AION (9–11), but the yield of a thrombophilic evaluation in patients with NA-AION, has not been proven (11, 12). Furthermore, homocysteine levels during the acute event appear to be similar in patients with NA-AION who are positive or negative for the C677T MTHFR mutation, which leads to elevated homocysteine levels (12), and a similar frequency of the MTHFR mutation was reported in patients with NA-AION and the general population (12). These findings suggest that homocysteine level and the C677T MTHFR polymorphism do not play a role in the occurrence of NA-AION.

Nocturnal hypotension has long been implicated as the “final insult” in compromised optic disk, leading to NA-AION. This assumption is based on studies showing that patients taking antihypertensive medications have a significantly lower mean nocturnal systolic blood pressure than normotensive individuals, and have a larger mean percentage decrease in systolic, diastolic, and mean blood pressures during the night (13). Others suggested that obstructive sleep apnea (OSA) may play a role in NA-AION owing to the relative ischemia that occurs during apneic episodes (14). Waller et al. (15) found that 71–89% of patients with NA-AION also had OSA, manifested by insomnia, snoring, and chronic fatigue. However, if nocturnal hypotension is indeed involved in NA-AION, the mechanism probably differs from that of OSA (7, 14, 16–18) given findings that OSA is not associated with a nocturnal decrease in blood pressure and the lack of a difference in the mean nocturnal decrease in blood pressure between patients with NA-AION and controls (19). Arda et al. (20) proposed that sleep apnea may not be a risk factor for NA-AION by itself but rather a contributory factor given its known deleterious effect on the vascular endothelium in diabetes, hypertension, and atherosclerosis. Although OSA can be treated with continuous positive airway pressure, this may not prevent NA-AION if the causes are multi- factorial (16).

### Optic disk appearance

A small cup-to-disk ratio (“disk-at-risk”) may be a risk factor for NA-AION. A study from the University of Iowa examining 608 consecutive NA-AION patients reported a significantly smaller cup-to-disk ratio than in the general population (21), supporting earlier findings (22). A postmortem study of the optic nerve 20 days after acute presentation of NA-AION yielded no correlation between the configuration of the infarct and the vascular territory (23). The morphology was not consistent with disease of the large or small vessels and seemed to represent a form of compartment syndrome. The authors postulated that in patients with a smaller disk, compartment syndrome secondary to the ONH edema compresses the vasculature of the ONH, leading to neuropathy. However, enlarged optic disk cup in NA-AION patient was reported (24).

### Medications

In patients with predisposing factors for NA-AION, phosphodiesterase-5 (PDE5) inhibitors used to treat erectile dysfunction, such as sildenafil, may disturb optic nerve autoregulation, leading to blood vessel dilatation and ONH edema (25). More data are still needed to corroborate this finding. Moreover, it is unclear if these effects are incidental or associated with their effects on the ocular circulation (26).

Optic neuropathy was also reported in 14 of 22 patients being treated with the antiarrhythmic drug amiodarone (27), typically bilateral. Upon discontinuation of amiodarone, only a few cases improved, all of whom had mild optic nerve dysfunction.

## Natural History of NA-AION

Up to 40% of NA-AION patients show spontaneous improvement in vision whereas 5–29% experience a continued deterioration over a few days or weeks (8, 28). Following this initial phase, further progression is unusual (28, 29). NA-AION recurs in the affected eye in 6% of patients within 2 years and in up to 7.6% within 3 years (30–32).

## Treatment of NA-AION

Because the pathologic mechanism of NA-AION is still unclear, suggestions for treatment are wide-ranging. None of the treatments attempted so far has proved effective in recovering the visual loss.

### Medications

Owing to its known anti-thrombotic activity, aspirin has been suggested for the treatment of patients after an initial event of NA-AION, in order to reduce the potential for hemostasis and inflammation and thereby prevent a recurrence in the fellow eye (33, 34). However, no significant long-term benefit was found. Nevertheless, given aspirin’s proven role in treating cardiovascular risk factors, many clinicians continue to recommend it for secondary prevention of NA-AION if not contraindicated (34).

In NA-AION, compression of the optic nerve vasculature by the edematous ONH is believed to increase the ischemic insult and worsen the visual prognosis. Therefore, attempts to reduce the ONH edema in the early stages of the disease with aggressive oral or intravitreally injected steroids were carried (2, 35, 36), but the results were disappointing. One study compared the outcome of 613 consecutive patients who voluntarily opted for either systemic corticosteroid treatment or no treatment (37). Among those with visual acuity up to 20/70 within 2 weeks of disease onset, rates of visual improvement after 6 months were 69.8% for the treated patients and 40.5% for the untreated patients. Among those with a moderate to severe initial visual field defect, corresponding rates were 40.1 and 24.5%. In another trial, patients who were treated with intravitreal triamcinolone to improve ONH edema in the acute phase of NA-AION exhibited more improvement in visual acuity and visual field at 6 months than patients who were not (38–40). However, none of these findings (37–40) have been confirmed in a large randomized controlled trial. Indeed, in a statement on the steroid controversy in NA-AION, researchers concluded that the data so far on intravitreal steroid treatment are at best anecdotal and at worst, potentially dangerous or misleading (41). Clinicians also need to take into account that corticosteroids may have significant systemic side effects, especially in elderly or vasculopathic patients (41).

The administration of bevacizumab, an anti-vascular endothelial growth factor antibody, to patients with NA-AION yielded satisfactory results in few cases (42). However, there are reports of NA-ION association with intravitreal bevacizumab use (43), and data from controlled randomized studies are lacking for this indication.

### Surgery

The ischemic optic neuropathy decompression study (IONDT), prompted by the compartment syndrome theory, indicated that decompression surgery was no better than careful follow-up. The 43% improvement in patients with careful follow-up as compared to 33% in the surgery group, with visual loss during the 6 months follow-up (12 vs. 24%, respectively), revealing that decompression surgery was harmful (44).

### Control of risk factors

Considering the poor results of treatments directed at the optic disk edema and inflammation associated with NA-ION, one of the best options at present is to control and correct any vasculopathic risk factors, such as severe hypertension, hyperglycemia, and hypercholesterolemia. Although these measures have not been proven to prevent a sequential acute event of NA-AION, they will likely improve the patients’ overall health.

## Animal Models of NA-AION: The Future Targets of Medical Treatment

Rodent and primate models of NA-AION have been developed to further understanding of the mechanisms underlying the disease (45–47). The models reproduce some signs of NA-AION across species, namely, ONH edema and loss of capillary perfusion in the optic nerve immediately behind the globe (but not in the retinal vessels), and all show long-term apoptosis of the retinal ganglion cells (RGCs) due to loss of axoplasmic transport in the damaged optic nerve (48, 49). The appearance of the fundus after injury in primates is very similar to that in humans, with ONH swelling and flame-like hemorrhages (49).

Rodent models of AION (rAION) have so far proved invaluable for testing potential novel treatments. Hyperbaric oxygen administered during the critical period immediately after induction of injury proved effective in preventing RGC loss (50). Intravenous or intravitreous prostaglandin J2 proffered almost equal protection when administered 5 h before rAION injury or immediately after, suggesting that it may have an even longer therapeutic window in humans in whom the injury develops more slowly, with episodes of recurring ONH edema (51, 52). Prostaglandin J2 is currently being tested in a primate model (Bernstein, 2014, personal communication). Others reported that subcutaneous granulocyte colony-stimulating factor (G-CSF) had both anti-apoptotic effects on the RGCs and anti-inflammatory effects on the optic nerve (53). Experiments in models of optic nerve crush (ONC), which induces severe edema in the ONH while allowing for examination of loss of microvascular perfusion and RGC death, show a protective effect of G-CSF when administered after injury (54, 55).

Clinical findings on the benefit of bevacizumab were supported in a murine model of ONC, showing that the drug preserved the integrity of the microvasculature (56). This apparently prevented post-ONC ischemia from spreading from the site of injury to the immediate retrobulbar portion of the nerve. Accordingly, experimental studies of intravitreal brimonidine, a peripherally acting α2 adrenergic agonist, reported an increase in short-term RGC survival after ONC (57). However, this effect is paradoxical: in glaucoma, brimonidine was found to reduce aqueous humor production by inducing vasoconstriction, whereas in rAION, the decreased RGC loss would be expected to be secondary to improved perfusion. Furthermore, a study conducted to evaluate the effects of brimonidine on AION revealed worse vision in those who received brimonidine (58). Therefore, brimonidine in the ONC setting may promote axonal growth (59) rather than affect vasoactivity, protect the RGCs in conditions of elevated intraocular pressure (60), and increase levels of brain-derived neurotrophic factor (BDNF) (61). There are as yet no studies of the effect of brimonidine on the oligodendrocytes, which are necessary for optic nerve axonal health and are directly injured by ONC. Brimonidine may be generally protective when axonal regrowth is required after rAION.

It is noteworthy, however, that findings of enhanced RGC survival after rAION (62) following pretreatment with brimonidine were not replicated in models of NA-AION (58, 63). Furthermore, the sudden onset of NA-AION and the typically delayed diagnosis make brimonidine pretreatment for NA-AION impractical, although translating from bench to clinic, brimonidine might be used prophylactically in the fellow unaffected eye of patients with NA-AION.

High doses of estrogen (64) were found to have no therapeutic value in rAION, compatible with the lack of gender specificity in NA-AION outcome, although women are considered protected until menopause.

Aside from G-CSF (54, 55), other exogenous compounds were found to have a protective effect against some aspects of ONC when administered after injury, including α-crystallin (65), BDNF (66–68), the PPARγ agonist pioglitazone (69), the sirtuin 1 (SIRT1) agonist resveratrol (70), pigment epithelial-derived growth factor (PEDF) (71), Toll-like receptor 4 (TLR4) antagonists (72), valproate (73), and endothelin-B antagonists (74). There is an even larger army of available drugs for potential pretreatment in ONC injury. Overall, they are directed against reducing inflammation or its signaling molecules, promoting survival or repair of damaged axons, or preserving patency of the blood supply. Almost none have been clinically tested.

## Summary

There is still no widely recognized treatment for NA-AION. Evidence from animal models of NA-AION generally suggests that reducing edema and inflammation in the acute phase might be effective. Intravitreal injections are becoming more commonly used.

The availability of drugs and the direct approach into the eye may be the best approach to therapy during the acute phase, allowing support for the injured RGCs. Improving RGC survival and reducing axonal damage after NA-AION is an active topic of investigation, directing future goals of neuronal regeneration or retinal neurogenesis. Cellular therapy, either by systemic transplantation or intravitreous administration, might become the next mode of treatment to improve visual outcome in NA-AION.

## Conflict of Interest Statement

The authors declare that the research was conducted in the absence of any commercial or financial relationships that could be construed as a potential conflict of interest.
